# Incarceration, menstruation and COVID-19: a viewpoint of the exacerbated inequalities and health disparities in South African correctional facilities

**DOI:** 10.1108/IJPH-05-2022-0033

**Published:** 2022-10-25

**Authors:** Janice Kathleen Moodley, Bianca Rochelle Parry, Marie Claire Van Hout

**Affiliations:** Department of Psychology, University of South Africa, Pretoria, South Africa; Chief Albert Luthuli Research Chair, University of South Africa, Pretoria, South Africa; Public Health Institute, Liverpool John Moores University, Liverpool, UK

**Keywords:** Menstrual hygiene management, Incarcerated women, COVID-19, South Africa

## Abstract

**Purpose:**

The menstrual health and menstrual hygiene management (MHM) of incarcerated women remains relatively low on the agenda of public health interventions globally, widening the inequitable access of incarcerated women to safe and readily available menstrual health products (MHP). The COVID-19 pandemic has adversely impacted on the MHM gains made in various development sectors in the global North and South, through its amplification of vulnerability for already at-risk populations. This is especially significant to developing countries such as South Africa where the incarcerated female population are an often-forgotten minority.

**Design/methodology/approach:**

This viewpoint highlights the ignominious silence of research and policy attention within the South African carceral context in addressing MHM. The ethical and political implications of such silences are unpacked by reviewing international and local literature that confront issues of inequality and equitable access to MHP and MHM resources within incarcerated contexts.

**Findings:**

Structural inequalities in various contexts around the world have exacerbated COVID-19 and MHM. Within the prison context in South Africa, women face multiple layers of discrimination and punishment that draw attention to the historical discourses of correctional facilities as a site of surveillance and discipline.

**Research limitations/implications:**

This study acknowledges that while this viewpoint is essential in rising awareness about gaps in literature, it is not empirical in nature.

**Practical implications:**

The authors believe that this viewpoint is essential in raising critical awareness on MHM in carceral facilities in South Africa. The authors hope to use this publication as the theoretical argument to pursue empirical research on MHM within carceral facilities in South Africa. The authors hope that this publication would provide the context for international and local funders, to assist in the empirical research, which aims to roll out sustainable MHP to incarcerated women in South Africa.

**Social implications:**

The authors believe that this viewpoint is the starting point in accelerating the roll out of sustainable MHP to incarcerated females in South Africa. These are females who are on the periphery of society that are in need of practical interventions. Publishing this viewpoint would provide the team with the credibility to apply for international and national funding to roll out sustainable solutions.

**Originality/value:**

It is hoped that the gaps in literature and nodes for social and human rights activism highlighted within this viewpoint establish the need for further participatory research, human rights advocacy and informed civic engagement to ensure the voices of these women and their basic human rights are upheld.

## Introduction

On March 11, 2022, the World Health Organization (WHO) declared COVID-19 as a pandemic (WHO, 2020). As a result, the vulnerability of at-risk populations heightened worldwide, and health inequalities for many vulnerable people and their communities have worsened (Jefferson *et al.*, 2021). One such vulnerable group is people who are deprived of their liberty in a variety of detention settings. The impact of COVID-19 within such detention settings, as described in developed countries (Crowe and Drew, 2021; Hawks *et al.*, 2020; Paynter *et al.*, 2021; Reinhart and Chen, 2021; Strassle and Berkman, 2020) and indeed in Africa (Jumbe *et al.*, 2022; Mhlanga-Gunda *et al.*, 2022; Muntingh, 2020; Nweze *et al.*, 2020; Van Hout, 2020a; Van Hout, 2020b; Van Hout *et al.*, 2022a; Van Hout *et al.*, 2022b), and South Africa (Van Hout and Wessels, 2021a) is the focus of our *Viewpoint.* Historical barriers to health in closed settings, such as overcrowding, poor hygiene facilities and resources, as well as poor ventilation, all of which intersecting with already poor menstrual health management conditions within detention facilities, will undoubtedly exacerbate the vulnerability of incarcerated women to COVID-19, with such environments conducive to spread of disease (Muntingh, 2020; Ohuabunwa and Spaulding, 2020).

Understanding the intersectional vulnerabilities that exist within detention settings, the United Nations (UN) has called for various measures to be initiated to ensure a decreased risk to public health within these facilities, including the early release of vulnerable incarcerated persons due to issues of over-crowding and having to eat, shower and toilet in communal areas (United Nations Office on Drugs and Crime, World Health Organization, UNAIDS and Office of the High Commissioner for Human Rights, 2020). Such measures are congruent to the normative UN standards of detention, for example the United Nations Standard Minimum Rules for the Treatment of Prisoners (the Nelson Mandela Rules) (United Nations Office on Drugs and Crime, 2016), the United Nations Rules for the Treatment of Women Prisoners and Non-custodial Measures for Women Offenders (the Bangkok Rules) (United Nations Office on Drugs and Crime, 2010), the United Nations Standard Minimum Rules for Non-custodial Measures (the Tokyo Rules) (UN, 1990), in addition to the African Charter on Human and People’s Rights (Organization of African Unity, 1981), and the nonbinding Robben Island Guidelines for the Prohibition and Prevention of Torture in Africa (Niyizurugero and Lessène, 2008).

At the global level both before and since the COVID-19, there is a wealth of evidence indicating continued health inequity of women in prisons, with their specific health needs routinely neglected and deprioritised. This is especially the case regarding their sexual and reproductive health (UNDOC, 2009). Lack of access to menstrual health products (MHP) in prison, such as sanitary towels is known as period poverty (IDPC, 2021; Penal Reform International, 2021).

Our *Viewpoint* concerns the right to menstrual hygiene management (MHM) in detention settings, with a focus on the African context and specifically South Africa. Globally, of the 11.5 million people deprived of their liberty, 741,000 are women (Penal Reform International, 2021). Over 1 million are detained in Africa (World Prison Brief, 2022), and in South Africa, women are a minority prison population, with 3 453 women incarcerated in the country (Department of Correctional Services [DCS], 2021; Van Hout and Wessels, 2021b).

## Menstruation health management: cultural dimensions, disparities, and COVID-19 impacts

Menstruation is an integral function of cis-women’s, trans and gender-nonconforming people’s health throughout their reproductive lifespan. Defined as the hygienic menstrual management of menstrual blood through the safe and hygienic use of, and disposal of, menstrual management materials (Kuhlmann *et al.*, 2017), MHM has become an emerging public health endeavour affecting approximately 50% of the world’s population who menstruate (Crawford *et al.*, 2019). According to the United Nations International Children’s Emergency Fund (UNICEF), approximately 800million women menstruate daily (UNICEF, 2015) and of the 1.8 billion females, non-binary, transgender men that menstruate, millions are unable to manage their menses hygienically and at their own discretion due to broader socio-economic disparities (Sumpter and Torondel, 2013) juxtaposed against cultural misconceptions (Padmanabhanunni *et al.*, 2018; Shannon *et al.*, 2021; Yamakoshi *et al.*, 2020) and taboos (Agyekum, 2002; O’Sullivan *et al.*, 2007; Strassmann, 1992).

Poor MHM is generally a consequence of poverty and deprivation (Bakibinga and Rukuba-Ngaiza, 2021; Hall, 2021; Rossouw and Ross, 2021). The impact of the COVID-19 pandemic on MHM remains largely unexplored (Spagnolo *et al.*, 2020). Beyond the COVID-19 devastations, we suspect that millions of women around the world have suffered and continue to suffer an accelerated untold erosion of basic human rights, bodily integrity and dignity due to the lack of access to adequate MHM (Ajari, 2020; Poague *et al.*, 2022; Salim and Salim, 2021). The vulnerability of economically and socially at-risk girls, women, trans and gender-nonconforming people who menstruate is potentially heightened during COVID-19 as a scarcity in sanitary products and adequate water, sanitation and hygiene facilities, disproportionately hampers their agency in managing their menstrual health hygienically at their own discretion (MacKinnon and Bremshey, 2020; Obani, 2021; Poague *et al.*, 2022).

Globally, the impact of poor MHM remains largely unknown (Sumpter and Torondel, 2013) due to the deeply historical and cultural construction of menstruation as an individual health concern framed within the private realm. As an individual and private concern, the solution for poor or inadequate MHM is framed as the responsibility of the individual, irrespective of socio-economic circumstances and despite very serious health consequences (Ajari, 2020; Carney, 2020; Harlow and Ephross, 1995; Torondel *et al.*, 2018). Over the past decade, menstrual health has increasingly become a global public health concern (Sommer *et al.*, 2015) that has been adopted as a human rights endeavour because of the social, political and economic disparities associated with MHM (Goldblatt and Steele, 2019).

Understanding the harmful gendered effects that women endure during times of pandemic and crisis, the World Bank, UNICEF and WHO, together with other health and gender advocacy agencies, have sounded the alarm by issuing briefs and recommendations aimed at assisting governments in creating gender conscientized health policies during the COVID-19 pandemic. Refrains such as *Periods don’t stop for pandemics* (World Bank, 2020) and *World can pause but periods cannot!* (Arora, 2020) highlight the urgency that is needed in managing MHM, particularly within contexts already lacking sustainable resources. This is echoed by the World Bank (2020) and their movement to end period poverty and period stigma by 2030. However, slow progress in development efforts hamper MHM in middle to low-income countries, in addition to poverty-stricken contexts in high income countries. 75% of households in low and middle-income countries have inadequate access to handwashing with soap (Eichelberger *et al.*, 2021) which is salient both to MHM and in stopping the transmission of the COVID-19 pandemic. Progress in within the African context, as in many other developmental contexts, have been hampered.

### Complexities of ensuing menstrual hygiene management in African prisons: pre and beyond COVID-19

Menstruation and other reproductive functions have been historically stigmatized as a mechanism of othering women, through its signification of difference between men and women (Frank, 2020; Van Hout & Crowley, 2021). Within the African context, social stigmatisations and cultural misunderstandings of menstruation have resulted in negative attitudes and experiences for women (Padmanabhanunni *et al.*, 2018; Shannon *et al.*, 2021). Researcher findings of such have been evidenced in Mali (Strassmann, 1992) and Ghana (Agyekum, 2002), as well as within the South African context (O’Sullivan *et al.*, 2007; Padmanabhanunni *et al.*, 2018). Framed as a “political silence”, (Goldblatt and Steele, 2019, p. 294), menstruation in contexts outside detention facilities are hushed because of the social and cultural stigmas associated with menstruation. Yet, much like women living on the outside, women within the carceral contexts face issues of menstrual equity at the intersection of discrimination. Within detention spaces globally, women lack autonomy over their own bodies, as they are reliant on the state to provide for their basic MHM needs (Weiss-Wolf, 2020). Indeed, the Bangkok rules acknowledge menstrual health, specifically in Rule 5 (United Nations Office on Drugs and Crime, 2010), which states that carceral centres are responsible for the provision of hygienic facilities and MHP, free from cost to women. Unfortunately, there is a lack of literature currently within the African and South Africa context, that explores how the impact of social and cultural stigmatisations, as well as the lack of bodily autonomy, manifest themselves in the menstruating experience within carceral facilities.

Even prior to the COVID-19 pandemic, the general and gendered health disparities faced by the incarcerated have largely escaped the priorities of global and African prison health agendas (Barberet and Jackson, 2017; Van Hout and Wessels, 2021b). For example, a systematic review which explored incarcerated female’s experiences of carceral health care in sub-Saharan Africa (SSA) over the past two decades, uncovered not only a dearth on incarcerated female experiences but also violations of human rights, coupled with poor health-care provision including lack of prisons system resourcing of sanitary products (Van Hout and Mhlanga-Gunda, 2018). The marginalized vulnerabilities of incarcerated women, though grossly unexplored, are expected to have exponentially heightened since the advent of the pandemic. Harsh and unexpected COVID-19 lockdown restrictions disrupting the supply of menstrual hygiene supplies, combined with pandemic induced economic strain on families, we suspect have invariably affected the MHM of incarcerated women leaving this already at-risk cohort at the mercy of overburdened state resources. In detention settings, deliberate or unintended restricted access to MHP and the inferior quality of MHP mean that incarcerated women may not have a sufficient supply of MHP per cycle (Carney, 2020).

Yet just as the pre-pandemic silence, the absence of scholarly work that critically engages with menstruation, internationally and locally, in detention facilities is distressing. Equally concerning are the immense barriers to access of researchers into prisons in Africa (Mhlanga-Gunda *et al.*, 2020). An opinion piece published in the Lancet, titled *What are the greatest health challenges facing people who are incarcerated? We need to ask them*, summarises the strides that need to be achieved in prioritising menstruation in detention facilities, when the only reference to gendered issues was listed as “gender-affirming care” (Berk *et al.*, 2021, p.703). It is within such narratives that the gross realities of women experience are neatly glossed over. The COVID-19 pandemic draws parallels to poor MHM. One a global catastrophic pandemic, one a seemingly hidden gendered issue, find commonality in that they both are symptomatic, and at the same time aggravated, by ailing health-care infrastructures. Both are an infringement on basic human rights to accessible and equitable health care heightened by pre-existing global health disparities. It is, therefore, unsurprising that the COVID-19 pandemic may amplify *the* “politics of health and health provision” (Jefferson *et al.*, 2021, p.149) of incarcerated women who are already marginalised and silenced within their contexts of restraint. This holds significance within most carceral contexts for example as documented in South Africa (Van Hout & Wessels, 2021b) where females remain a forgotten minority.

### South Africa: women in detention spaces and right to menstrual health

Prisons in SSA have seen an increase in the incarcerated female population in recent years (Penal Reform International, 2016; Van Hout and Mhlanga-Gunda, 2018; Walmsley, 2017). South Africa has one of the largest prison populations on the African continent (World Prison Brief, 2022), with the latest Department of Correctional Services (DCS) report for 2020/2021 indicating that there are currently 140,948 individuals incarcerated in South Africa. Of this total, 137 495 are men and 3,453 are women, making up a small percentage of 2.45% (DCS, 2021). As one of the countries who are a signatory for the United Nations Standard Minimum Rules for Non-Custodial Measures, the DCS in South Africa follows the delegated guidelines and minimum standards for the provision of health care services of the men and women remanded to their custody (DCS, 2016). Recognition of these rights and responsibilities are protected through local regulations as well and include the Correctional Services Act (DCS, 1998) and the White Paper on Corrections in South Africa (DCS, 2005). Yet there are long standing concerns surrounding the incarcerated population’s well-being, including overcrowding, poor nutrition and deteriorating facilities, all of which impact the physical and mental health of incarcerated populations (Agboola, 2016; Ajari, 2020; Van Hout and Mhlanga-Gunda, 2018; Van Hout and Wessels, 2021b).

There is a resounding silence within the South African context in highlighting and prioritizing menstrual health equity within incarcerated contexts. Despite the advances that have been made in prioritizing gender, the MHM within correctional facilities remains largely unexplored within the South Africa context (Artz *et al.*, 2012; Artz and Hoffman-Wanderer, 2017; du Preez, 2008; Haffejee *et al.*, 2005; Hopkins, 2016; Luyt and du Preez, 2010). The veiled secrecy that envelopes most carceral contexts in South Africa means that deliberate menstrual discrimination based on multiple intersectionalities goes unchallenged (Carney, 2020). As reported by Van Hout and Wessels (2021a), the longstanding and precarious situation of women in detention settings in South Africa since post-apartheid timeframes needs to be highlighted, with the visibility of women enhanced, particularly with regards to poor living conditions (including a lack of availability of menstrual products), reasonable and safe accommodation and protection from custodial violence. It is therefore unsurprising that the COVID-19 pandemic would raise the alarm as a correctional health crisis, particularly when considering the devastation that HIV/AIDS and tuberculosis has wrought on the South African carceral community. Despite this, media coverage and academic attention surrounding COVID-19 in corrections has been framed as gender-neutral, with incarcerated women all but invisible and the impact of the pandemic on their lives ignored (Ellis, 2020; Van Hout and Wessels, 2021b). The unique health needs of incarcerated female population in an already-overburdened system that is overcrowded and unhygienic, places women at great risk of having their health needs relegated and neglected.

### Gendered impacts of COVID-19 for incarcerated South African women’s menstrual hygiene management

Unfortunately, as the female population comprise only a fraction of the general incarcerated population in South Africa, there exists a research vacuum and narrative silence around the unique situation posed for incarcerated women, before and during the advent of the COVID-19 pandemic (Agboola, 2016; Mussell *et al.*, 2020; Padmanabhanunni *et al.*, 2018). To date, only one study has addressed the menstrual health narratives of a portion of the incarcerated in South Africa. In the context of a broader study of lived experience, Agboola (2016) explicates the narratives of 10 previously incarcerated women, who discussed the conditions of their carceral MHM. Their accounts corroborate previous findings from research in South African correctional facilities, where access to health-care services is limited, as are necessary general hygiene provisors such as like soap and water, all of which are exacerbated by high levels of overcrowding. For women, the situation is far more dire. It is evident that the incarcerated female population have complex health needs with disproportionate rates of underlying health conditions when compared to women in general, and so necessitates the understanding that they often have greater gender-specific, primary healthcare needs in comparison to their male counterparts, a reality that is particularly evident with regards to menstruation (Agboola, 2016). Both local (Gender, Health and Justice Research Unit, 2012) and international (Corston, 2007) research studies indicates that, on average, incarcerated women are issued with two sanitary pads for each day that they are menstruating, at the cost of the state. However, this resulted in a policing of periods, where women were forced to provide evidence of soiled sanitary towels to correctional staff before replacements were issued (Agboola, 2016).

Even in ordinary circumstances incarcerated women’s health-care needs necessitate unique undertakings in the male dominated carceral environment, but when resources are diverted into emergency health provisions for COVID-19, it is not unlikely that access to reproductive health services behind bars will be impacted (Rope, 2020). Women have special hygiene requirements which correctional facility authorities are obliged to provide for, along with hygienic menstrual material disposal. Reports during the pandemic have been that globally, lockdown efforts have resulted in limited delivery and access to sanitary products (Barnes, 2020; Sommer *et al.*, 2020; UNICEF and UNFPA, 2020). Women in correctional facilities have had to go without sanitary products during COVID-19 crisis management lockdowns, as MHP such as tampons or menstrual cups are often provided by external support networks like charities or family members, who are no longer able to visit as access to prisons by external visitors has been prohibited. Although tampons and other vital MHP may be available from the correctional commissary, they are often sold at inflated prices which can be cost-prohibitive (Ellis, 2020). “Family and friends visiting prisoners are in many ways the lifeblood of the prison, bringing not only human interaction and contact with the outside world but also resources such as cash, food, bedding, toiletries and so forth” (Muntingh, 2020, p.5). For vulnerable and marginalized women, including the incarcerated population, the pandemic crisis may result in menstruation becoming a time of deprivation and stigma, when faced shortages and reduced privacy under lockdown (UNICEF and UNFPA, 2020).

### A “new normal” and incarcerated women agency in menstrual hygiene management

As stated, within the detention space, MHM becomes a public matter, rather than occupying its usual space in the private lives of women. Whereas prior to incarceration they were in the sole care of their menstruation and menstrual symptoms, once in a correctional facility, this fundamental aspect of the women experience becomes a public affair. Of course, this impacts their embodied agency within the constraints of their carceral surroundings, which form part of a penal system designed with the male body in mind, a space where women’s bodies and needs are invisible (Bostock, 2020). The conceptualisation of such experiences is manifested in the term *period poverty*, which Bostock (2020) denotes as a form of biopower, where menstrual inequality in corrections and the restriction of sanitary products is used to gain control of women through their biology. In response to social and physical distancing measures and lockdowns to manage the COVID-19 pandemic, issues of carceral accountability and oversight increase as do concerns with incarcerated women’s agency, privacy, autonomy, hygiene, and self-sufficiency. “The new restrictions allow for less accountability and more isolation than we have seen in decades” (Mussell *et al.*, 2020, p. 5). To complicate matters further incarcerated women usually come from marginalized and disadvantaged backgrounds, characterized by histories of substance abuse, violence, physical and sexual abuse, all of which exacerbate physical and mental health problems (Agboola, 2016; Parry, 2020; van den Bergh *et al.*, 2011).

Even prior to the challenges imposed by the COVID-19 pandemic, incarcerated women found monthly menstrual management, as well as accompanying myths and taboos, led to high levels of menstrual distress, particularly prevalent within the South African cultural milieu (O’Sullivan *et al.*, 2007; Padmanabhanunni *et al.*, 2018; Scorgie *et al.*, 2016). Many vulnerable women state that they have a lack of understanding of the menstrual cycle and are unable to function normally as they are physically and mentally weaker during menstruation, experiencing issues with bodily cleanliness, feeling “dirty” during their menstrual period, as well as “vulnerable” as they believed it was a time of “openness” of the body with “a susceptibility to infection and illness” (Smith, 2009, p.5). The sexual and disgust connotations of menstruation, coupled its secretive demeanour, mean that poor menstrual management resources and misinformation result in its monthly onset ensuing a fraught and anxious time for women. Therefore, raising awareness regarding menstruation and hygienic practices, as largely a neglected area in terms of research, is imperative to dignified menstrual health practices for vulnerable women (Sumpter and Torondel, 2013). The persistence of shame and stigma regarding menstruation requires far more than the provision of sanitary products, it requires the sustained effort and intervention in developing the incarcerated women self-esteem and agency concerning their bodies in order to improve their menstrual health practices (Geismar, 2018). Unfortunately, such bodily empowerment seems unlikely in a carceral environment in the grips of COVID-19, where basic health interventions of sanitation and social distancing are hampered by lacking resources and failing infrastructure. Moreover, the withdrawal or lapse of incarcerated women’s reproductive health care and its diversion into COVID-19 crisis health care treats menstruation as a commodity rather than a basic human right, further exacerbating period poverty in female correctional centres.

The serious dearth of information on the experience of menstruation and of menstrual symptoms of the South African incarcerated community (Gender, Health and Justice Research Unit, 2012) necessitates the undertaking of academic interest and research to better understand the nature of their menstrual health management and its impact on their lives, both inside and outside of correctional facilities. Even before the advent of COVID-19 there was an increasing need to understand the incarcerated community’s MHM and period poverty, alongside studies concerning the unique experiences of transgender and non-binary menstruating people in corrections (Chrisler *et al.*, 2016; Lane *et al.*, 2021). “It is essential to understand the unique and diverse oppressions faced surrounding period poverty to ensure appropriate and proportionate activism, legislation and improvements for menstruating people in prisons” (Bostock, 2020, p. 7). South Africa was the first African country to adopt a constitution that explicitly prohibits discrimination on the basis of gender, sex and sexual orientation (amongst other categories) (Section 9 of the South African Constitution). The Equality Court judgement of September v Subramoney was the first of its kind in South Africa (and Africa) by tackling the equality rights of transgender prisoners, and rights to dignified detention and reasonable accommodation (Van Hout, 2022a). By analogy this case could leverage for greater rights assurances of menstruating women, and women in general in South Africa’s prisons. Additionally, the 2020 judgement of Sonke Gender Justice NPS v President of the Republic of South Africa is of further relevance to the situation of women menstruating in prison, and, held that section 7(2) of the Constitution required the State to take reasonable steps to protect the rights of incarcerated persons (Van Hout, 2020b).

To this end, the COVID-19 pandemic offers an opportunity for the DCS to fully integrate an empathetic and rights-based approach that is more in line with the South African government’s Department of Women’s (2019) Sanitary Dignity Implementation Framework for the provision of sanitary dignity. Minister Ronald Lamola (Lamola, 2020) issued a press release assuring the United Nations that South Africa would adhere more closely to the Mandela rules following the pandemic. If our *new normal* during and after the COVID-19 pandemic can be orientated towards reducing inequalities and increasing empowerment for women, particularly vulnerable and women like those incarcerated, then MHM must be part of that conversation. Any new practices adopted in light of the pandemic should be sustainable and instituted long term, setting a precedent going forward and becoming entrenched practice (Prais, 2020). Although enabling every women in South African to manage their menstruation safely and comfortably is not a simple undertaking, especially in the carceral environment, establishing menstrual health management as an actionable public health issue is imperative (Geismar, 2018). Such adopted practises and policies can do much to establish and maintain meaningful development around menstruation and empowerment in the post COVID-19 era to come.

## Concluding remarks

Our Viewpoint highlights the potential equality and basic human rights violations of menstruating women in South African prisons pre-COVID and beyond. Extant jurisprudence can be leveraged to support strategic public litigation, along with various efforts to sensitise government, promote civil society activism and encourage further research to inform policy and practice which sufficiently uphold the rights of women. South Africa has ratified the Optional Protocol to the UN Convention Against Torture, and national preventive mechanisms are advised to fully consider inspections regarding menstrual management provisions in South Africans prison system going forward.

Structural inequalities in various contexts around the world have exacerbated COVID-19 and MHM disparities within historical contexts of deprivation. This has very real continuing health consequences for the girls, women, non-binary, and transgender men who lack access to the resources and facilities needed to safely manage their monthly cycle at their own discretion. MHM disparities require multisectoral collaboration between public heath, legal, human rights, and carceral contexts for menstrual equity and human rights issues to advance. It is essential for governments, big businesses and development organisations and projects, to find innovative and cost-effective strategies for meeting the crisis response to the COVID-19 pandemic, but also in achieving a sustainable supply of MHM to those inside and outside of carceral facilities.

Within the prison context in South Africa, women face multiple layers of discrimination and punishment that draw attention to the historical discourses of correctional facilities as a site of punishment, surveillance, and discipline. Too often, the voices of those most vulnerable are missing from commentaries and activism on menstrual health issues. There is a growing need for transparency within carceral facilities that research can provide by exploring the lived experiences of women and corrections officers in managing MHM. The COVID-19 pandemic presents unique challenges to access to carceral facilities that need to be confronted. Restricted research access to carceral facilities could signify that any unequitable and in humane retreatment of incarcerated women may go unopposed. Additionally, the absence in menstrual health literature, particularly within the African context, means that the intersection of health disparities and racial discrimination that the COVID-19 pandemic has highlighted, remains unknown and, therefore, unchallenged with carceral contexts, indicating the need for future research prioritisation.

Finally, in this *Viewpoint*, we acknowledge that menstruation is not an exclusive feature of the female body since non-binary and transgender men may also menstruate. There is currently a punishing silence in international and national literature that seeks to understand the lived menstrual experiences of non-binary and transgender men both inside and outside of carceral facilities. The structural restrictions of their menstrual bodies go unchallenged in contexts where historical constructions of masculinity pervade. The is a necessary area of social, legal, ethical and research development in MHM.
